# Multi‐Objective Inverse Design of a Dual‐Narrowband Thermal Emitter for Optical Gas Sensing

**DOI:** 10.1002/nap2.70295

**Published:** 2026-09-18

**Authors:** Xiaowen Li, Zilin Zhang, Jiazheng Tang, Sheng Liu, Jie Sheng, Qionghui Chang, Xuyang Zhang, Yuchuan Shao, Zhengji Wen, Feng Liu

**Affiliations:** ^1^ Department of Physics Shanghai Normal University Shanghai China; ^2^ Laboratory of Thin Film Optics Key Laboratory of Materials for High‐Power Laser Shanghai Institute of Optics and Fine Mechanics Chinese Academy of Sciences Shanghai China

**Keywords:** aperiodic multilayer, dual‐narrowband thermal emitter, inverse design, optical gas sensing

## Abstract

Spectrally selective narrowband thermal emitters with high‐temperature stability are critical infrared sources for advanced gas sensing. However, conventional Tamm‐plasmon narrowband thermal emitters are commonly implemented as distributed Bragg reflectors on thin metal layers, which can restrict spectral tunability and complementary metal–oxide–semiconductor (CMOS) compatibility. Here, we propose a cost‐effective, CMOS‐compatible, lithography‐free, wafer‐scale aperiodic dual‐narrowband thermal emitter. Optimized via a multi‐objective inverse design framework, the emitter consists of an aperiodic Si/SiO_2_ dielectric multilayer deposited on a TiN refractory conductive ceramic film. Experimental results demonstrate that the fabricated sample exhibits two selective emission peaks at 3.26 and 6.30 μm, matching the characteristic absorption bands of CH_4_ and NO_2_, respectively. In addition, the emitter maintains its dual‐narrowband spectral response after heating to 693 K. In practical gas‐sensing tests, the dual‐narrowband emitter achieved 12.2‐ and 13.5‐fold improvements in relative sensitivity for CH_4_ and NO_2_ detection, respectively, compared with a conventional broadband blackbody source. This work provides a scalable refractory infrared source for spectrally selective optical gas sensing.

## Graphical abstract

A lithography‐free, wafer‐scale dual‐narrowband thermal emitter is developed using a multi‐objective inverse design framework. It comprises an aperiodic Si/SiO_2_ dielectric multilayer deposited on a TiN refractory conductive ceramic film. Its dual emission peaks match characteristic absorption bands of CH_4_ and NO_2_ and enhance responses to both gases, providing a scalable refractory infrared source for spectrally selective optical gas sensing.

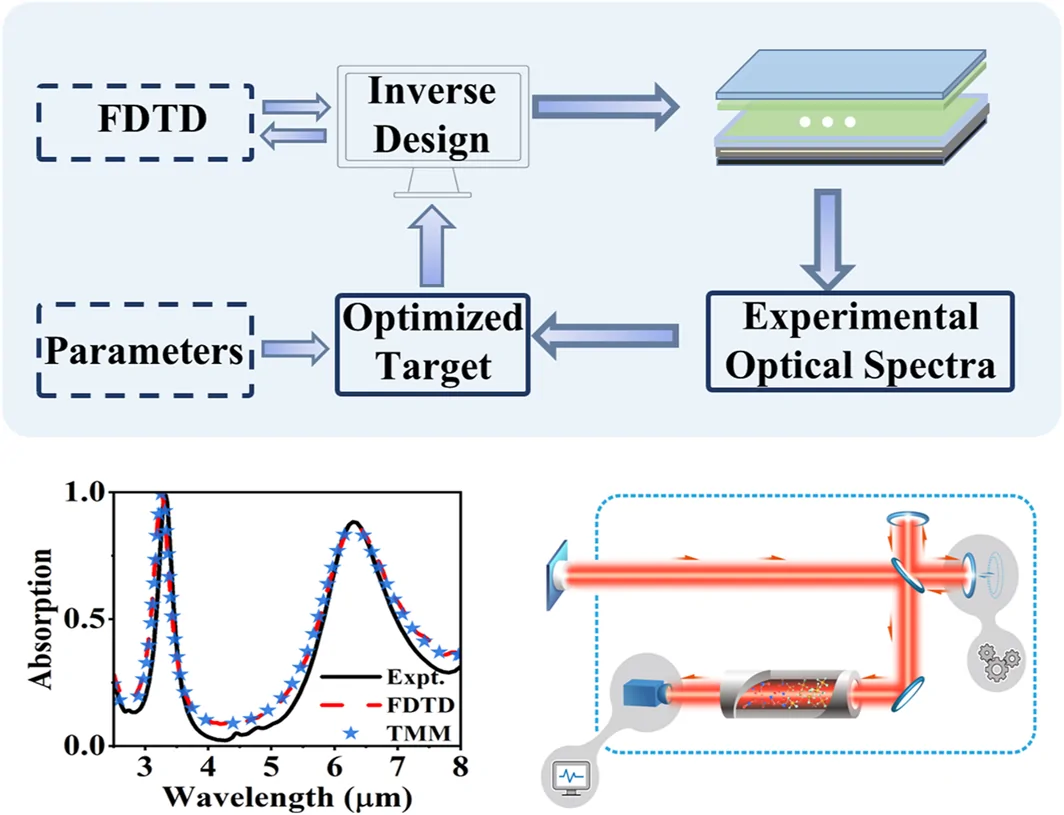

## 1 Introduction

Thermal radiation governed by Planck's law is broadband, which limits its efficiency in applications requiring high spectral specificity [1]. In fields such as passive radiative cooling [2, 3, 4, 5, 6, 7], thermophotovoltaics (TPV) [8, 9, 10], infrared multilevel encryption [11], infrared camouflage [12, 13, 14, 15], and non‐dispersive infrared (NDIR) optical gas sensing [16, 17], the emission of photons outside a narrowly defined target band constitutes a parasitic energy loss and leads to severe signal‐to‐noise degradation. Therefore, it is highly desirable to develop artificially engineered emitters that can overcome these broadband limitations, enabling precise control over the emission bandwidth and angular distribution to satisfy specific functional requirements. Narrowband thermal emission has been achieved using photonic crystals [18, 19, 20, 21, 22], surface relief gratings [23, 24], optical nano‐antennas [25, 26], and metamaterial or metasurface emitters [27, 28]. Beyond spectral selectivity, polarization control has also become an important direction in thermal emission engineering. By combining local control of the polarization state with nonlocal high‐Q resonances, thermal metasurfaces can produce narrowband and directional emission with prescribed linear, elliptical, or circular polarization which offers new opportunities for polarization resolved spectroscopy and chiral sensing [29, 30, 31, 32, 33, 34]. However, these structures are often limited by either complex manufacturing processes or poor thermal stability. To deal with these problems, an alternative method is to use multilayer thin‐film structures [5, 12, 35, 36, 37]. Such structures can support optical resonances such as Tamm plasmon polaritons (TPPs) or Fabry–Perot (FP) cavity modes [38, 39], which can be engineered to realize narrowband thermal emission. Unlike patterned nanostructures which often rely on noble metals that are incompatible with standard complementary metal‐oxide‐semiconductor (CMOS) processing, planar multilayers are entirely lithography‐free and can be rapidly fabricated using standard physical vapor deposition (PVD) techniques. Moreover, the multilayer dielectric stack designed for thermal emission is usually arranged in a periodic fashion, and the thickness of each dielectric layer is set to one‐quarter of the operating wavelength. Advances in multi‐objective optimization have made the inverse design of complex aperiodic multilayer structures possible [40, 41, 42].

Gas detection has been implemented using a variety of methods, including electrochemical sensors, catalytic sensors, metal‐oxide semiconductor sensors, and optical spectroscopic sensors [43, 44, 45]. Among various gas‐sensing technologies, optical gas sensors offer advantages such as high sensitivity, long‐term stability, fast response, and suitability for real‐time and in situ detection [46]. Since each gas in nature absorbs electromagnetic waves due to the vibrations of its molecules, and the vibrational frequency of the gas molecules corresponds to the gas's characteristic absorption wavelength [47, 48], thermal emission becomes highly desirable for gas sensing when its narrowband resonances match the molecular absorption signatures of the target gases [17, 49]. As a result, utilizing narrowband thermal emitters as light sources can simplify the overall sensor architecture while enhancing detection sensitivity, spectral selectivity, and energy efficiency compared with conventional broadband sources. Most previous studies on narrowband thermal emitters have focused on single‐gas detection systems with only one resonant wavelength [35]. In practical environmental monitoring, however, simultaneous detection of multiple hazardous or greenhouse gases is often required. This work focuses on two critical environmental pollutants [50]: methane (CH_4_), a potent greenhouse gas and the primary component of natural gas [51, 52], and nitrogen dioxide (NO_2_), a toxic pollutant associated with high‐temperature combustion and traffic emissions [53, 54]. Because the selected CH_4_ and NO_2_ sensing windows are centered at 3.26 and 6.30 μm, respectively, a dual‐narrowband thermal emitter operating at these wavelengths is desirable for multiplexed gas sensing.

In this work, we present the theoretical analysis, inverse design framework, physical fabrication process, and experimental validation of a wafer‐scale, CMOS‐compatible, dual‐peak mid‐infrared narrowband thermal emitter tailored for NDIR sensing of CH_4_ and NO_2_. The proposed structure comprises an optimized aperiodic sequence of continuous silicon (Si) and silicon dioxide (SiO_2_) layers deposited on a titanium nitride (TiN) refractory conductive ceramic film. TiN is used as the conventional metallic back reflector mainly due to three factors: (1) it circumvents the spectral tunability limitations imposed by fixed plasma frequency constraints, (2) it ensures complete compatibility with standard CMOS fabrication processes, and (3) as a refractory material, it exhibits excellent thermal stability and can sustain the high‐temperature operation required for practical thermal‐emitter applications. The geometric parameters are engineered using a multi‐objective particle swarm optimization (MOPSO) algorithm [55, 56, 57], integrated with high‐efficiency transfer matrix method (TMM) analytical solvers and finite‐difference time‐domain (FDTD) numerical analyses [58, 59]. The fabricated emitter maintains stable high‐temperature emission and exhibits pronounced spectral‐directional selectivity. Finally, we integrate the optimized structure into a gas‐sensing platform and demonstrate order‐of‐magnitude improvements in the relative sensitivity for both target gases compared with a conventional blackbody reference emitter.

## 2 Results and Discussion

### 2.1 The Closed‐Loop Optimization Framework and Inverse Design Process

Designing an aperiodic multilayer thermal emitter that supports two narrowband resonances is a high‐dimensional optimization problem. Traditional forward‐design strategies become inefficient when both the material sequence and the thickness of each discrete layer vary independently. Therefore, a highly robust closed‐loop inverse‐design framework was developed, as illustrated in Figure 1a. The workflow starts from the target resonant wavelengths (3.26 and 6.30 μm), manufacturing constraints, and candidate material boundaries. To simulate the optical properties of these candidate structures, we evaluated both the analytical transfer matrix method (TMM) and the numerical finite‐difference time‐domain (FDTD) method. Since the TMM offers exceptional computational efficiency, it was selected as the primary electromagnetic solver driving the multi‐objective optimization process. FDTD was then reserved for the cross‐validation method to verify the absorption results. In addition, Fourier‐transform infrared spectroscopy (FTIR) data measured from fabricated materials were fed back into the model to calibrate the optical‐constant database and improve robustness against fabrication‐induced deviations.

**FIGURE 1:**
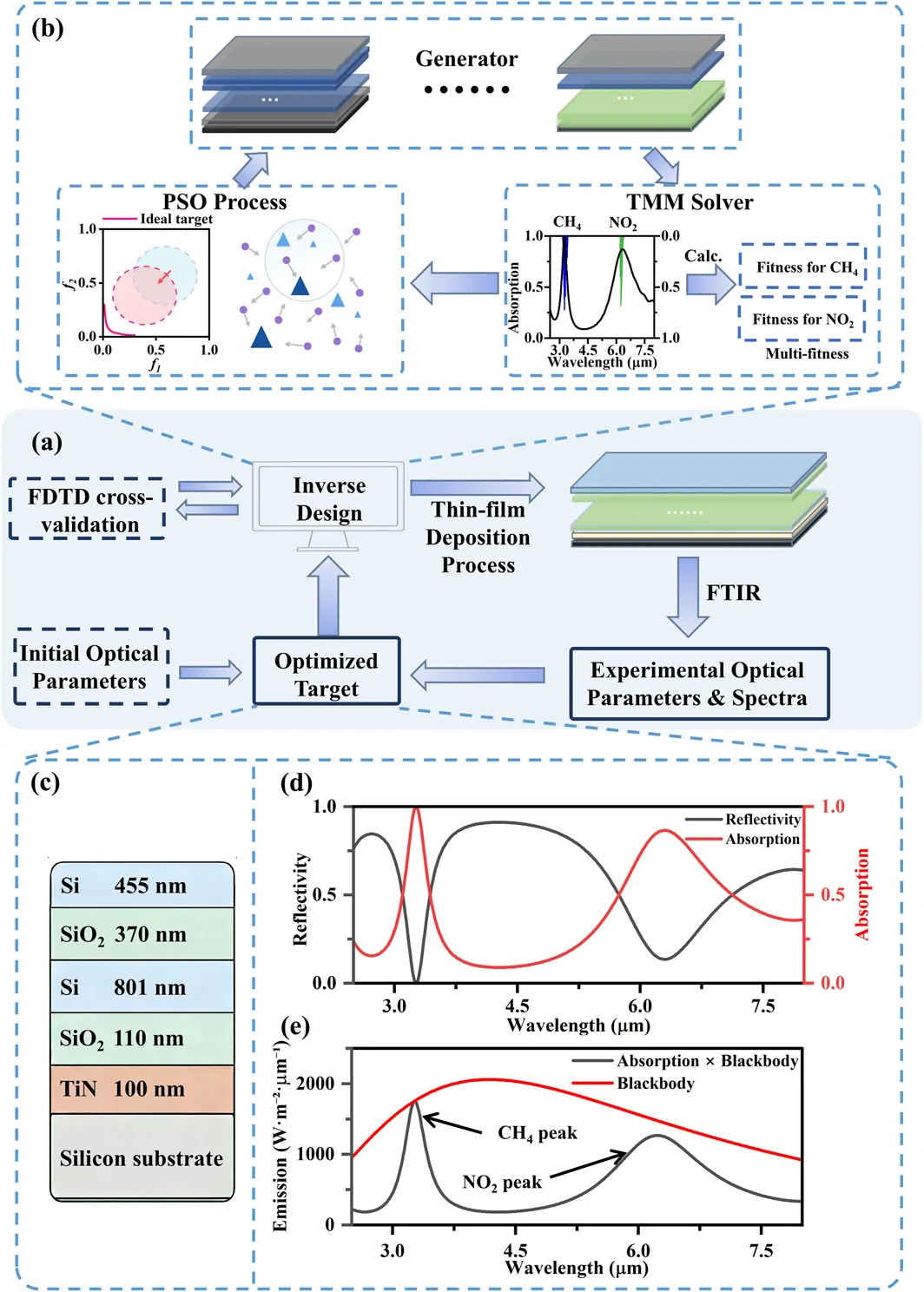
Schematic of the inverse‐design framework and algorithmic performance. (a) Closed‐loop inverse design workflow. The process integrates the transfer matrix method (TMM) with multi‐objective optimization and uses finite‐difference time‐domain (FDTD) simulations for cross‐validation. A critical experimental feedback loop (bottom path) employs Fourier transform infrared spectroscopy (FTIR) data from fabricated samples to calibrate the optical parameters of the material. (b) Schematic of the MOPSO process used to design the dual‐narrowband thermal emitter. (c) Final optimized structure obtained by inverse design. (d) Absorption and reflectance spectra of the optimized structure, showing agreement with the dual‐wavelength target. (e) Calculated emission spectrum obtained by multiplying the absorptivity by the blackbody spectrum at 693 K.

To optimize the required structure, we employed a multi‐objective particle swarm optimization (MOPSO) algorithm, whose basic workflow is shown in Figure 1b. In each iteration, candidate structures are evaluated by the TMM solver, compared with the target spectrum, and then updated through the MOPSO procedure until convergence. For this dual‐peak application, the initial material library comprised CMOS‐compatible high‐index candidates (Nb_2_O_5_, Si, TiN, Bi_2_Te_3_) and low‐index candidates (SiO_2_, Al_2_O_3_) [41]. Based on preliminary evaluations, the optimal material subset was determined to be alternating layers of silicon (Si) and silicon dioxide (SiO_2_) deposited on a 100 nm thick titanium nitride (TiN) film. This optically thick TiN layer functions as an opaque conductive refractory ceramic back‐reflector, reducing transmission to zero. To quantify the spectral performance of candidate structures and guide the swarm, a composite fitness function (FF) was defined in Equation (1):

(1) $$FF=\omega_{\text{peak}}\cdot S_{\text{peak}}+\omega_{\text{contrast}}\cdot S_{\text{contrast}}+\omega_{\text{bandwidth}}\cdot S_{\text{bandwidth}}$$

where $S_{\text{peak}}$, $S_{\text{contrast}}$, and $S_{\text{bandwidth}}$ quantify the target dual‐band peak absorption, the spectral selectivity between target and non‐target regions, and the bandwidth of the target bands, respectively. The weights are set to $\omega_{\text{peak}}=0.3$, $\omega_{\text{contrast}}=0.6$, $\omega_{\text{bandwidth}}=0.1$. The strong prioritization of spectral contrast is tailored specifically for NDIR gas sensing because it minimizes the out‐of‐band emission.

After 100 iterations of the MOPSO optimization loop, the particle swarm converged to the final optimized structure shown in Figure 1c. The corresponding theoretical absorption and emission spectra are presented in Figure 1d,e. The calculated emission peaks are located at 3.26 and 6.30 μm, matching the selected CH_4_ and NO_2_ sensing windows, respectively.

### 2.2 Electromagnetic Field Distributions and Resonant Absorption Mechanisms

To illustrate the working mechanisms of the optimized structure, rigorous FDTD simulations were performed under a normal incident plane wave. Figure 2 presents the electric field intensity (∣E∣), magnetic field intensity (∣H∣), and power loss density ($P_{a}$). The top row (Figure 2a–c) corresponds to the CH_4_ resonant peak at 3.26 μm, whereas the bottom row (Figure 2d–f) corresponds to the NO_2_ resonant peak at 6.30 μm.

**FIGURE 2:**
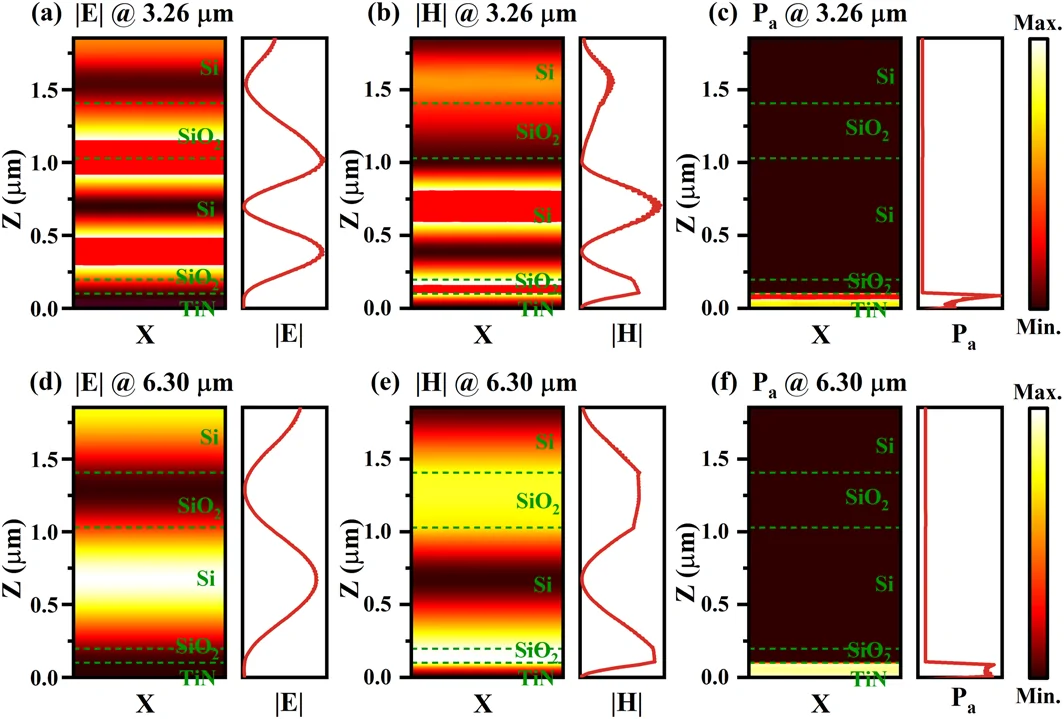
FDTD simulations of the two‐dimensional (2D) electric field intensity (∣E∣), magnetic field intensity (∣H∣), and power loss density (Pa) inside the optimized structure under normal incidence. Red lines show the corresponding one‐dimensional (1D) cross‐section plots. The top row (a, b, c) corresponds to the short‐wave resonant peak at 3.26 μm, whereas the bottom row (d, e, f) corresponds to the long‐wave resonant peak at 6.30 μm.

As shown in the electric and magnetic field profiles (Figure 2a,b,d,e), the resonant fields are strongly confined near the interface between the Si/SiO_2_ multilayer and the TiN interface and penetrate into the adjacent aperiodic dielectric stack [60]. This field‐localization behavior indicates the excitation of Tamm plasmon polariton (TPP)‐like resonant modes supported by the asymmetric Si/SiO_2_ dielectric structure and the TiN back reflector. In this configuration, the aperiodic Si/SiO_2_ stack serves as an inverse‐designed wavelength‐selective dielectric reflector with a Bragg‐reflector‐like response, whereas TiN provides the metallic boundary needed to support the interface modes. The nonuniform layer sequence creates different wavelength‐dependent confinement conditions, enabling two spectrally distinct TPP‐like resonances at 3.26 and 6.30 μm.

To further clarify the absorption mechanism, we calculated the spatial distributions of the time‐averaged power loss density as shown in Figure 2c,f. The photon energy is not absorbed within the highly transparent Si/SiO_2_ dielectrics. Instead, the loss is concentrated in the TiN conductive ceramic layer. Therefore, the TiN film functions both as an optically thick back reflector and as the dominant absorption layer. This boundary‐localized loss distribution indicates that the resonant absorption is associated with TPP‐like modes coupled to the asymmetric multilayer cavity.

### 2.3 Sample Fabrication and Spectral‐Directional Characterization

After numerical validation of the optimized design, the structure was fabricated using the physical vapor deposition workflow, as schematically illustrated in Figure 3a. The process started with a polished silicon wafer substrate. The initial stage utilized ion beam sputtering (IBS) to deposit a 100 nm thick layer of TiN. Following TiN, the dielectric multilayer stack (the optimized Si and SiO_2_ sequences) was deposited via electron beam deposition (EBD), allowing for precise monitoring of individual layer thicknesses.

**FIGURE 3:**
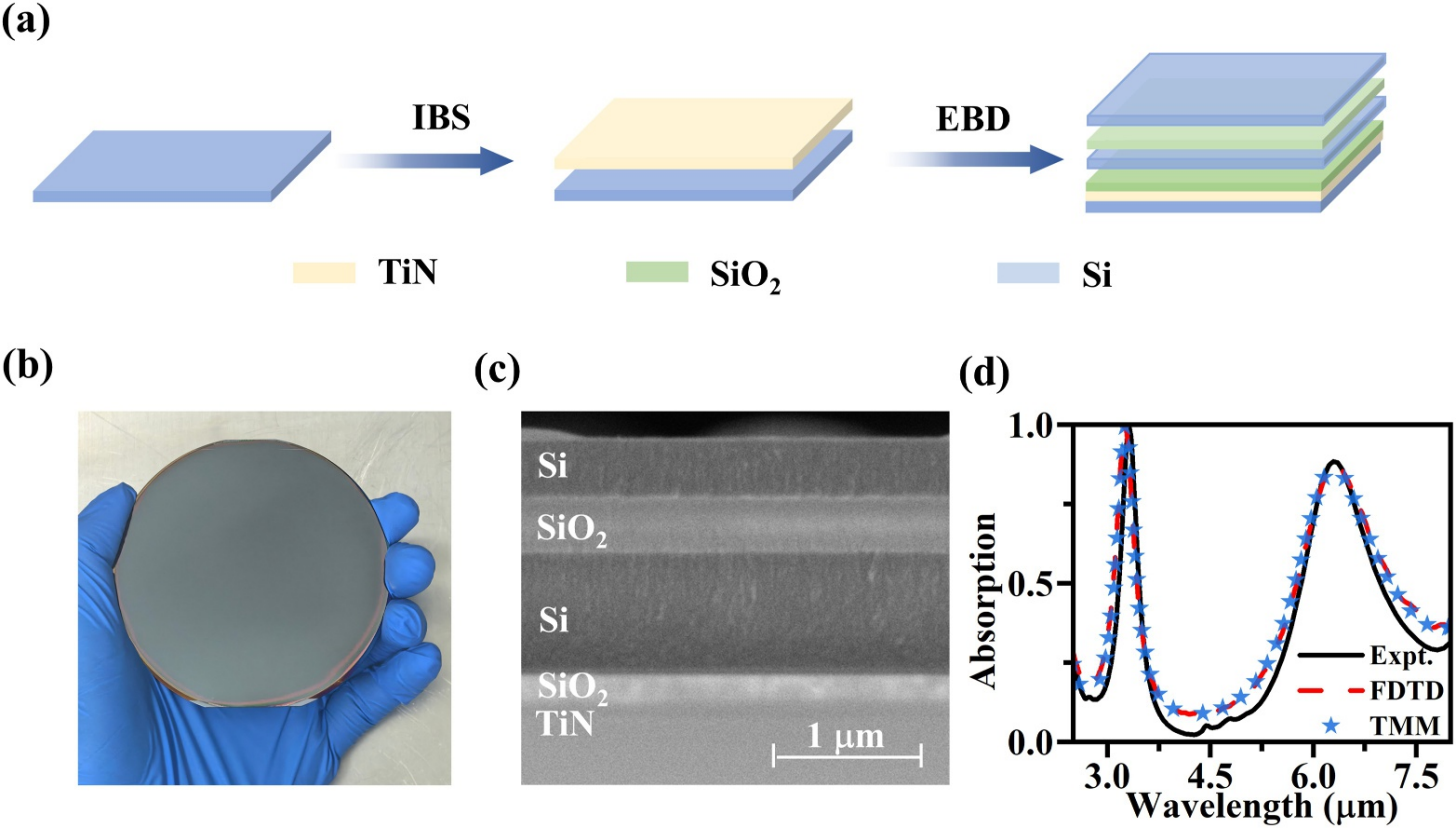
Fabrication process and structural characterization of the multilayer thermal emitter. (a) Schematic illustration of the hybrid deposition workflow, consisting of ion‐beam sputtering (IBS) for the TiN layer and electron beam deposition (EBD) for the dielectric stack (Si/SiO_2_). (b) Photograph of the fabricated large‐area sample with a diameter of 10 cm. (c) Cross‐sectional scanning electron microscopy (SEM) images of the fabricated structure. (d) Comparison between the experimentally measured near‐normal‐incidence absorption spectrum and the calculated performance at normal incidence using TMM and FDTD. The experimental absorption spectrum (black line) agrees well with both the FDTD simulation (red dashed line) and TMM theoretical calculation (blue star markers).

The fabrication process produced a 10‐cm‐diameter (4‐inch) sample, as shown in Figure 3b. To confirm the actual thicknesses of the deposited layers and the fidelity of the multilayer architecture, cross‐sectional scanning electron microscopy (SEM) was performed. Figure 3c shows clearly resolved Si, SiO_2_, and TiN layers with nearly parallel interfaces, indicating that the fabricated structure is consistent with the design. The spectral performance was then validated by absorption measurements. As shown in Figure 3d, the experimental spectrum agrees closely with both the TMM design and the FDTD simulation, confirming the accuracy of the inverse design and fabrication process. The dual‐narrowband emission peaks are matched to the characteristic absorption bands of NO_2_ and CH_4_, thereby enhancing optical gas‐sensing performance.

### 2.4 Spectral‐Directional and Temperature Characterization

For NDIR sensing, the spectral‐directional emission properties of the emitter must be comprehensively characterized. Unlike conventional sources, the intensity and peak wavelength of narrowband thermal emission from resonant structures are highly sensitive to the angle of observation. We therefore investigated the absorption of the fabricated sample under varied incidence angles and polarization states. Theoretical contour plots mapping the infrared absorption as a function of wavelength and incident angle were generated. Figure 4a–c show the theoretical absorptivity under *s*‐polarization (*s*‐pol.), *p*‐polarization (*p*‐pol.), and unpolarized (unpol.) incidence states, respectively.

**FIGURE 4:**
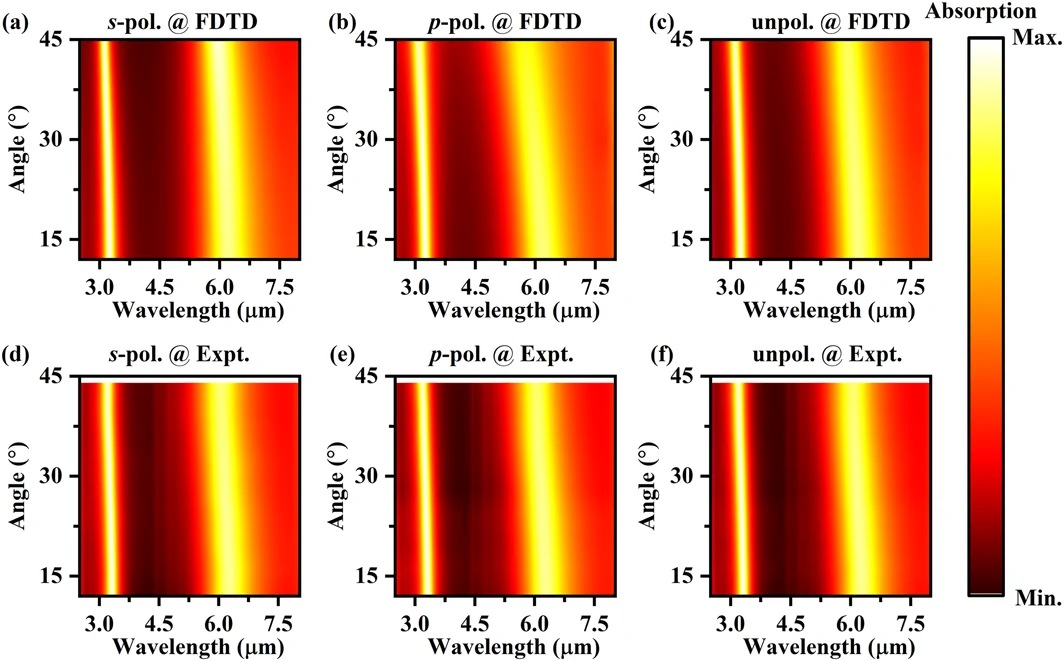
Spectral‐directional absorption characterization. (a–c) Calculated infrared absorption across wavelength and incidence‐angle space for *s*‐polarized (*s*‐pol.) (a), *p*‐polarized (*p*‐pol.) (b), and unpolarized (unpol.) incidence (c). (d–f) Experimentally measured infrared absorption under the corresponding polarization states.

Subsequently, experimental angle‐resolved reflection measurements were performed to validate the calculations. Figure 4d–f show the experimental absorption corresponding to the same polarization states as Figure 4a–c. The experimental and theoretical results all show good agreement. Furthermore, we detected the emission change due to different observation angles, which is shown in Figure 5a–c, exhibiting the exact same trend as the absorption data. The emission spectra follow the same angular trend as the absorption data, in agreement with Kirchhoff's law of thermal radiation. To further evaluate the spectral‐directional properties, the radiation intensities at the 3.26 and 6.30 μm peaks are shown in detail in Figure 5d–i. These figures clearly indicate that the emissivity spectra recorded at negative viewing angles act as perfect, mirror‐symmetrical extensions of the positive angle spectra due to the isotropic nature of the planar structure. The strongest absorption and emission are concentrated near the surface normal, mainly within 0°–15°, indicating that the proposed emitter radiates dual‐narrowband infrared light in a well‐defined angular range suitable for use as an infrared source and multi‐species gas‐sensing applications.

**FIGURE 5:**
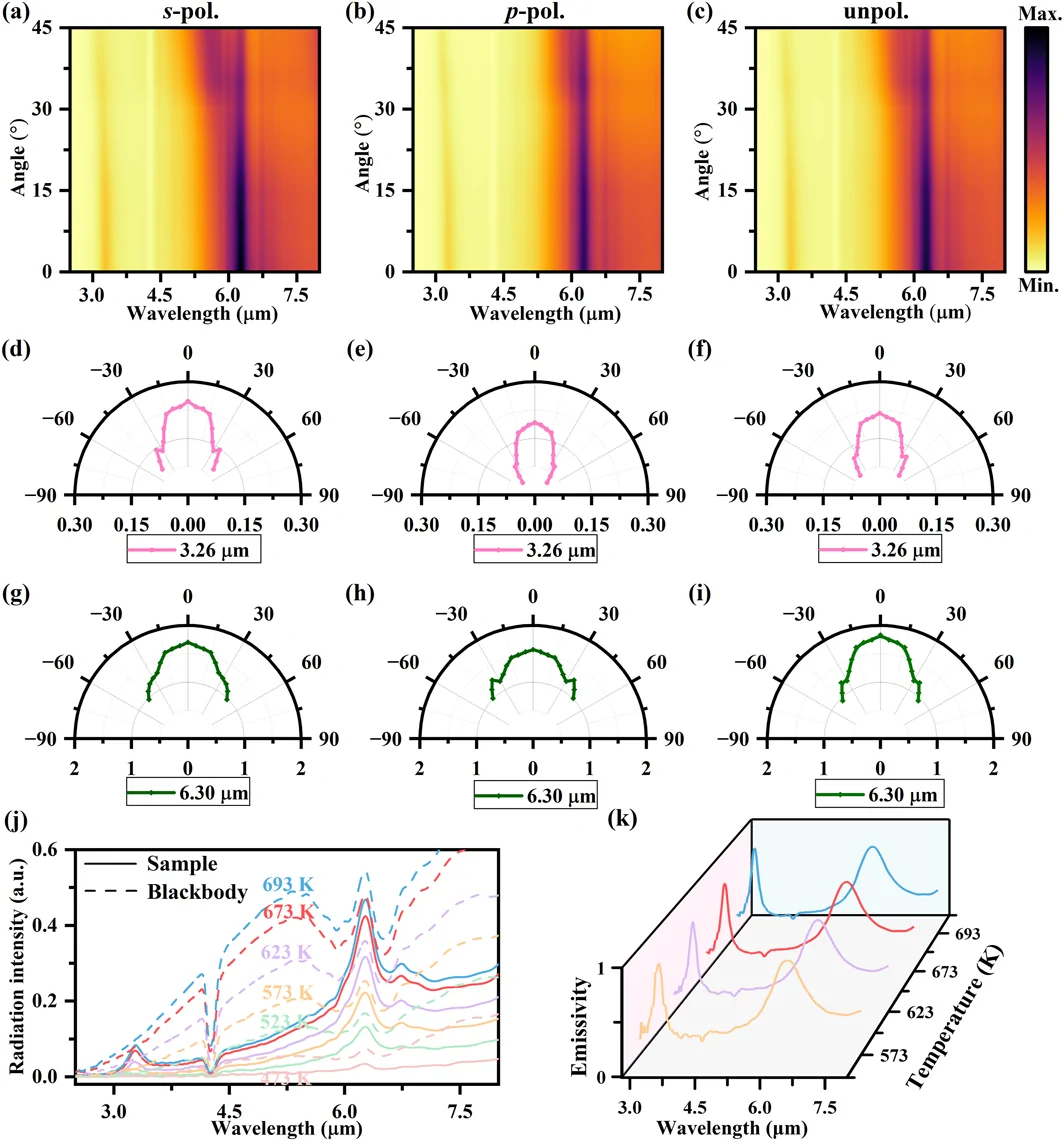
Spectral‐directional emission and high‐temperature stability. (a–c) Experimental infrared emission as a function of wavelength and incident angle under *s*‐polarized (*s*‐pol.) (a), *p*‐polarized (*p*‐pol.) (b), and unpolarized (unpol.) emission (c). (d–i) Corresponding angular radiation distributions at 3.26 and 6.30 μm for the three polarized cases. (j) Experimentally measured radiation intensity spectra for the fabricated emitter sample (solid lines) and blackbody reference (dashed lines) at six temperatures from 473 to 693 K. The radiation valley near 4.2 μm originates from atmospheric CO_2_ absorption. (k) Corresponding normalized emissivity in (j).

The operational viability of the emitter also depends on high‐temperature stability. To evaluate this stability, the fabricated sample was heated and its emission spectra were monitored with an FTIR spectrometer. Figure 5j,k show that the structure maintains the dual‐narrowband thermal radiation peaks at 3.26 and 6.30 μm during heating from 473 to 693 K. The radiation intensity was directly measured by FTIR, and the normalized emissivity was obtained by dividing the sample radiation intensity by that of a blackbody reference under the same temperature conditions.

### 2.5 Gas‐Sensing Application and Performance Evaluation

As illustrated in Figure 6a, the two emission peaks of the fabricated thermal emitter match the characteristic molecular absorption bands of methane (CH_4_, shaded blue) and nitrogen dioxide (NO_2_, shaded green). In contrast, a standard blackbody reference operating at the same temperature exhibits a broad emission profile with minor fluctuations caused by atmospheric and water‐vapor absorption. To quantify practical gas sensing performance, a dual‐channel non‐dispersive infrared gas detection test platform was constructed. Figure 6b shows the device schematic, integrating the fabricated dual‐peak thermal emitter with a sealed gas cell and an FTIR spectrometer. Here, the FTIR was used for spectral validation and performance evaluation. As the CH_4_ and NO_2_ measurements were performed separately without mixed‐gas testing, the present results demonstrate the spectrally selective response of the emitter to each target gas under single‐gas conditions rather than simultaneous mixed‐gas detection. In a compact NDIR device, the FTIR can be replaced by calibrated fixed‐band detection channels centered at 3.26 and 6.30 μm, following previously demonstrated nonscanning and filter‐free NDIR architectures based on narrowband thermal emitters and wavelength‐selective detector arrays [49, 61, 62]. For each target gas, a certified 2000 ppm stock gas was used. Each concentration was prepared in an independent measurement run after the gas cell was returned to the air baseline. In each run, 5, 10, 15, 20, 25, or 30 mL of stock gas was introduced into the 126 mL gas cell, producing nominal concentrations of 79, 158, 238, 318, 397, and 476 ppm, respectively.

**FIGURE 6:**
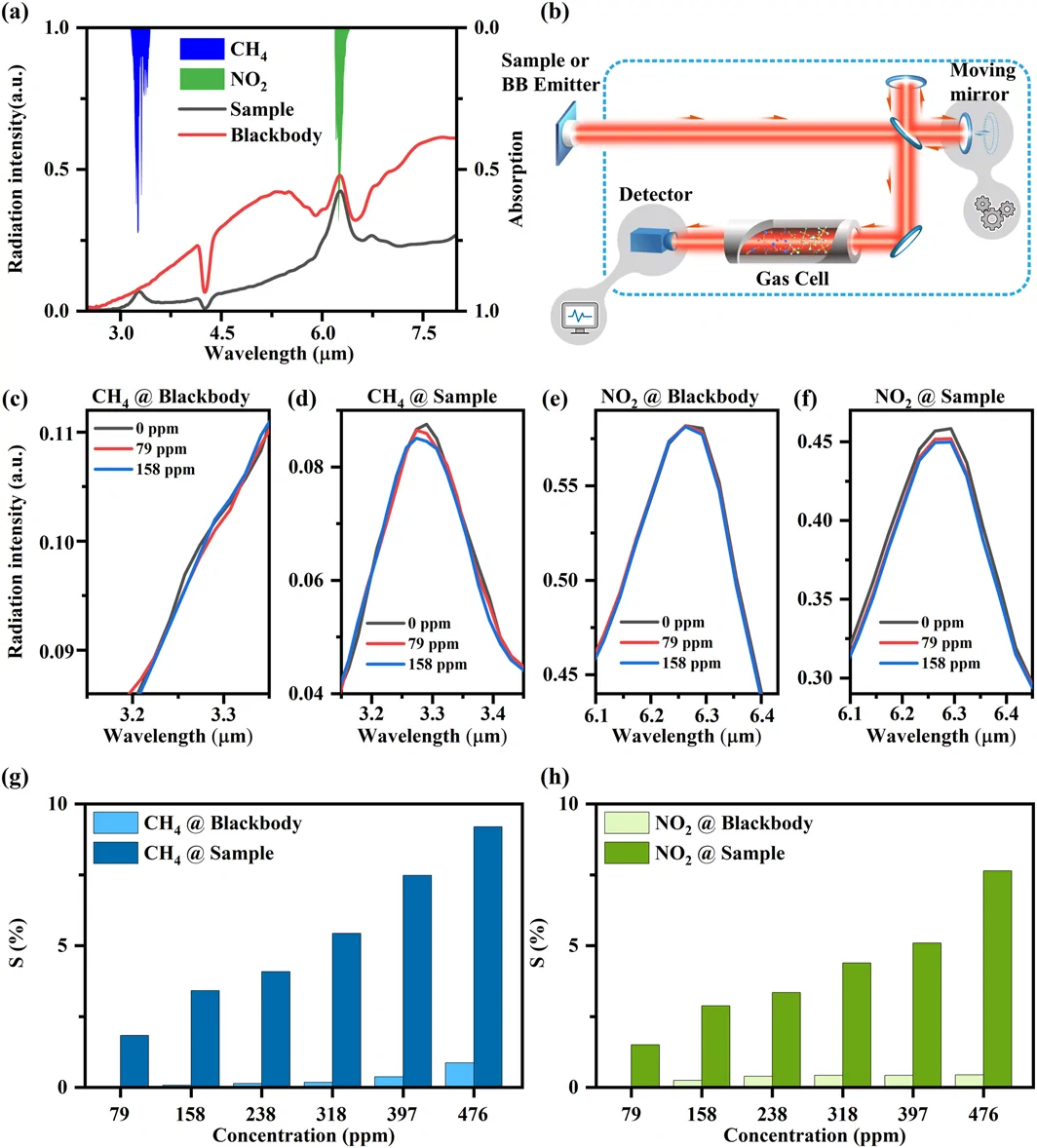
Gas‐sensing tests and performance evaluation. (a) Illustration of the thermal emitter's main emission peaks with the characteristic absorption bands of CH_4_ and NO_2_. (b) Schematic of the NDIR dual‐channel gas detection system. (c–f) Magnified radiation intensity spectra with the applied gas concentrations for the four cases (CH_4_ @ Blackbody, CH_4_ @ Sample, NO_2_ @ Blackbody, NO_2_ @ Sample). (g, h) Normalized sensitivity of the thermal emitter compared with the blackbody source.

The measured attenuation spectra are presented in Figure 6c–f. Figure 6c,d respectively show the attenuation of the emission intensity of the blackbody and the thermal emitter as the CH_4_ concentration increases. It is clearly observed that the emission intensity of the thermal emitter decreases significantly with CH_4_ concentration changes, whereas the attenuation of the blackbody emission remains much weaker. The NO_2_ measurements exhibit a similar response trend, as shown in Figure 6e,f. To quantitatively evaluate this detection performance and isolate the target gas response from external parasitic variables, a normalized sensitivity metric (S) was defined in Equation (2):

(2) $$S=\frac{I_{0}-I}{I_{0}}$$

where $I_{0}$ and I denote the measured intensities before and after introduction of the target gas, respectively. Figure 6g,h show that across the entire experimental concentration range, the thermal emitter exhibited a stable concentration dependent increase in normalized sensitivity for both gases. The emitter's relative sensitivity is an order of magnitude higher than the blackbody.

To compare the sensing performance quantitatively, the relative sensitivity (RS) was defined as the slope of the linear relationship between the normalized sensitivity (S) and the specific gas concentration (C), formulated as $RS=\frac{dS}{dC}\times 100\%\;\left(\%/\text{ppm}\right)$. Linear regression fitting was applied to determine the RS values. For CH_4_ detection, the standard blackbody source yielded an RS of $1.51\times 10^{-3}\;\%/\text{ppm}$, whereas the engineered thermal radiator achieved an outstanding *RS* of $1.84\times 10^{-2}\%/\text{ppm}$, representing an approximate 12.2‐fold enhancement. For NO_2_ detection, the standard blackbody produced an *RS* of $1.05\times 10^{-3}$%/ppm, whereas the thermal radiator reached $1.42\times 10^{-2}$%/ppm, corresponding to a 13.5‐fold improvement. These tests show that concentrating thermal radiation within the selected absorption bands enhanced the responses to both gases, demonstrating the emitter's potential for optical gas sensing.

## 3 Conclusion

In summary, we have designed and experimentally demonstrated a cost‐effective, CMOS‐compatible, lithography‐free, wafer‐scale dual‐narrowband infrared thermal emitter based on an aperiodic Si/SiO_2_ dielectric multilayer deposited on a TiN refractory conductive ceramic film. Derived via a multi‐objective inverse design framework, the emitter exhibits selective narrowband thermal emissivity peaks at 3.26 and 6.30 μm and maintains stable spectral performance up to 693 K. The fabricated sample was further integrated into a gas‐sensing platform for CH_4_ and NO_2_ detection, where it achieved 12.2‐ and 13.5‐fold improvements in relative sensitivity, respectively, compared with a conventional blackbody emitter. We anticipate that this scalable nanophotonic approach could support the development of high‐performance infrared sources for precision optical gas sensing and environmental monitoring and high‐temperature thermophotovoltaics.

## 4 Experimental Procedures

### 4.1 Numerical Simulations

The ideal spectrum was evaluated using three‐dimensional full‐wave simulations implemented with the finite‐difference time‐domain (FDTD) method. The electromagnetic response of the structure under planewave excitation was calculated within a three‐dimensional computational domain. Periodic boundary conditions were applied along both in‐plane directions (x and y). Reflected and transmitted powers were measured by monitors positioned on the incidence and exit sides of the structure, respectively. Moreover, separate two‐dimensional monitors depicted the spatial distributions of the electromagnetic fields.

### 4.2 Sample Fabrication

Commercially polished silicon (Si) substrates were cleaned sequentially with acetone and deionized water, then dried under flowing nitrogen. A 100 nm thick titanium nitride (TiN) refractory conductive ceramic film was deposited on the Si substrate using an ion‐beam sputtering system (Bühler Leybold Optics ARES 1110) to ensure high crystallinity and sub‐nanometer interfacial roughness. After that, the TiN coated film was placed into the high‐vacuum chamber of an electron beam evaporation system to deposit the Si and SiO_2_ dielectric layers. The evaporation rates and exact individual physical thicknesses of the Si and SiO_2_ films were precisely monitored and controlled in real time by an in situ optical and quartz crystal microbalance system. Finally, cross‐sectional images of the corresponding fabricated samples were characterized by scanning electron microscopy (SEM; Carl Zeiss Auriga).

### 4.3 Optical Characterization

The angle‐resolved reflectance spectra of the dual‐narrowband emitter sample were characterized using an FTIR spectrometer (Thermo Fisher Scientific iS50) equipped with a reflection module that allowed incident angles from 10° to 60°. Before each set of reflectance measurements, a background spectrum was recorded using a silver mirror under the same measurement conditions. This reference spectrum was automatically applied by the FTIR software (OMNIC) to correct the measured reflectance spectra. Because the optically thick TiN layer suppresses transmission, the absorption spectra were calculated as (A=1−R) from the background‐corrected reflectance spectra. Angle‐resolved emission spectra were recorded using the same iS50 FTIR spectrometer operated in the external emission mode. Unlike the reflectance mode, this emission configuration does not provide an equivalent silver mirror based automatic background correction function. The sample was placed on a temperature controlled hot plate and heated at 60°C/min. After reaching 693 K, the sample was allowed to equilibrate for approximately 10 min before spectral acquisition.

### 4.4 Gas‐Sensing Tests

During each gas‐cell measurement, radiation from the fabricated emitter was directed through the gas cell before spectral acquisition. Gas spectra were recorded using an FTIR spectrometer (Thermo Fisher Scientific iS50). In this experiment, a static gas distribution method was adopted. Certified CH_4_ and NO_2_ stock gases at 2000 ppm were used. Each concentration was prepared in a separate measurement run using a 126 mL gas cell. Before each run, the gas cell was returned to the air baseline. The injections were not cumulative. In each run, 5, 10, 15, 20, 25, or 30 mL of stock gas was introduced, whereas an equal volume of air was displaced to maintain constant pressure. After each gas injection, the sealed cell was held for 100 s to allow the gas to diffuse and the concentration distribution to stabilize before the spectrum was collected. The concentration was calculated using $C_{f}=\frac{V_{a}C_{s}}{V_{c}}$, where $V_{a}$ is the injected stock‐gas volume, $C_{s}$ is the stock‐gas concentration (2000 ppm), and $V_{c}$ is the gas‐cell volume (126 mL). The resulting nominal concentrations were 79, 158, 238, 318, 397, and 476 ppm. The NO_2_ samples were prepared using the same procedure. For comparison, a blackbody was used as the external light source, and the same measurements were repeated.

## Author Contributions

**Xiaowen Li:** resources, writing – review and editing, writing – original draft, conceptualization, methodology, software, data curation, supervision, project administration, visualization, funding acquisition, investigation, validation, formal analysis. **Zilin Zhang:** methodology, software, data curation, writing – review and editing, writing – original draft, validation, visualization, formal analysis. **Jiazheng Tang:** methodology, validation, visualization, writing – review and editing, software, formal analysis, conceptualization. **Sheng Liu:** conceptualization, writing – review and editing, methodology, software, data curation, formal analysis. **Jie Sheng:** conceptualization, methodology, formal analysis, software, visualization, writing – review and editing, data curation. **Qionghui Chang:** data curation, formal analysis, validation, visualization, writing – review and editing, methodology. **Xuyang Zhang:** methodology, validation, visualization, writing – review and editing, formal analysis, data curation. **Yuchuan Shao:** methodology, visualization, validation, writing – review and editing, conceptualization, software. **Zhengji Wen:** writing – review and editing, visualization, writing – original draft, conceptualization, methodology, data curation, supervision, formal analysis, funding acquisition, validation, resources, project administration. **Feng Liu:** conceptualization, investigation, funding acquisition, writing – review and editing, methodology, software, data curation, resources, supervision, formal analysis, project administration, validation, visualization.

## Funding

This research was supported by National Natural Science Foundation of China (12141303, 12373099, 12504376); Shanghai Science and Technology Committee (24ZR1475000); Young Elite Scientists Sponsorship Program by CAST (YESS20220355)

## Conflicts of Interest

The authors declare no conflicts of interest.

## Data Availability

Data underlying the results presented in this paper are not publicly available at this time but may be obtained from the authors upon reasonable request.
